# Changes in Macronutrients during Dieting Lead to Weight Cycling and Metabolic Complications in Mouse Model

**DOI:** 10.3390/nu16050646

**Published:** 2024-02-25

**Authors:** Anouk Charlot, Anthony Bringolf, Léa Debrut, Joris Mallard, Anne-Laure Charles, Emilie Crouchet, Delphine Duteil, Bernard Geny, Joffrey Zoll

**Affiliations:** 1 Biomedicine Research Center of Strasbourg (CRBS), UR 3072, “Mitochondrie, Stress Oxydant et Plasticité Musculaire”, University of Strasbourg, 67000 Strasbourg, France; anouk.charlot@etu.unistra.fr (A.C.);; 2 Faculty of Sport Sciences, University of Strasbourg, 67000 Strasbourg, France; 3 CNRS, University of Strasbourg, Inserm, IGBMC UMR 7104-UMR-S 1258, 67400 Illkirch, France; 4 Institute of Cancerology Strasbourg Europe (ICANS), 67200 Strasbourg, France; 5 Faculty of Medicine, University of Strasbourg, 67000 Strasbourg, France; 6 Institut de Recherche sur les Maladies Virales et Hépatiques UMR_S1110, University of Strasbourg, Inserm, 67000 Strasbourg, France; 7 Service de Physiologie et Explorations Fonctionnelles, University Hospital of Strasbourg, 67091 Strasbourg, France

**Keywords:** low-carbohydrate high-fat diet, obesity, non-alcoholic fatty liver disease, weight cycling, nutrition, carbohydrates

## Abstract

Weight cycling is a major challenge in obesity management. Caloric restriction is known to promote this phenomenon, but the impact of macronutrient changes during dieting remains unclear. This study aimed to determine the role of macronutrient changes in weight maintenance without caloric restriction by alternating between two hypercaloric diets: a high-carbohydrate, high-fat Western diet (WD) and a low-carbohydrate, high-fat diet (LCHDF). Obesity was induced in 8-week-old C57BL/6 male mice by 10 weeks of WD feeding. Then, the mice were subjected to 12 weeks of LCHFD interspersed with WD (I-WD), 3 periods of 2-week LCHFD followed by 2 periods of 3-week WD, or 12 weeks of continuous WD (C-WD). C-WD and I-WD mice were compared to standard diet (SD) mice. In the I-WD group, each LCHFD period decreased weight gain, but mice regained weight after WD resumption. I-WD mice exhibited obesity, dyslipidemia, and glucose intolerance, similarly to the C-WD mice. I-WD mice also developed nonalcoholic steatohepatitis, associated with an increase in type-III collagen gene expression and a decrease in FGF21 protein levels, in comparison with SD. I-WD mice developed weight cycling despite maintaining a high caloric consumption, suggesting that changes in macronutrients during dieting are also a trigger of weight regain.

## 1. Introduction

Obesity has increased over time and is now considered a pandemic; this is a major challenge of the 21st century. Obesity incidence is increased by several factors, such as unhealthy eating behaviors, including the consumption of a Western diet (WD), which is defined as a hypercaloric diet rich in fats, refined carbohydrates, and added sugar [1]. Since obesity is associated with health complications, such as type 2 diabetes and nonalcoholic fatty liver disease, curative strategies are needed [2]. Among them, dietary approaches are based on a reduction in caloric intake and lipid consumption to induce a negative energy balance between energy intake and expenditure, which is supposed to result in weight loss [3]. However, these strategies are frequently inefficient considering that 80% of obese individuals who lose more than 10% of their body weight tend to regain their weight within one year [4].

This phenomenon, characterized by weight loss and weight regain, is called “yo-yo dieting” or “weight cycling” and induces metabolic disturbances and complication developments [5], such as an increased risk of future weight gain or abdominal fat [6,7], type 2 diabetes [8,9], nonalcoholic fatty liver disease (NAFLD) [10], or metabolic syndrome [11].

NAFLD is the most prevalent form of liver disease and is characterized by a lipid accumulation that can progress to steatosis, inflammation, and fibrosis that can be identified by histological staining [12]. Weight cycling, and particularly weight regain, exacerbate liver weight gain and steatosis [13]. The hepatic damage induced by weight cycling seems to involve alterations in fatty acid metabolism, including an increase in the expression of the de novo lipogenesis enzymes, such as fatty acid synthase (FASN) and acetyl-coA carboxylase (ACC), and collagen markers like collagen I (Col1a1) and Collagen III (Col1a3) [14,15]. NAFLD is also associated with an upregulation of the transcriptional factors, notably the peroxisome proliferator-activated receptor γ (PPARγ) and the CCAAT/enhancer binding protein α (C/EBPα), which promote lipid storage, while fatty acid oxidation becomes insufficient in clearing the liver of lipids [16,17].

Weight cycling may be explained by the response to hypocaloric diets, which increase hunger and the reward value of food, leading to increased food intake after the diet ends [18,19]. In addition, caloric restriction prompts the body to reduce energy expenditure, including exercise energy expenditure, thermogenesis, and the resting metabolic rate, to conserve energy and restore homeostasis [20,21,22,23,24]. These metabolic adaptations to resist weight loss are maintained for several years after weight loss and impair the long-term maintenance of weight loss when the diet is interrupted [25,26].

In the face of the inefficiency of caloric restriction in weight loss maintenance, a hypercaloric diet, based on changes in macronutrient distribution without caloric restriction, offers a promising strategy for preventing obesity complications. The hypercaloric low-carbohydrate, high-fat diet (LCHFD) reduces weight gain and insulin levels and improves glucose tolerance in diet-induced obese mice [27,28,29]. In clinical trials, an LCHFD induced weight loss and decreased blood glucose levels, steatosis, and aspartate aminotransferase (AST) and alanine aminotransferase (ALT) levels in patients with obesity [30,31,32,33,34].

However, while caloric restriction has been described as a trigger of weight cycling development, the impact of macronutrient changes without caloric intake modification during dieting remains unclear, and the molecular pathways involved in the establishment of weight cycling are poorly understood. Therefore, we investigated the effects of 12 weeks of an LCHFD interspersed with a WD in an obese mouse model from a systemic to a molecular level. The aim of the present study was to determine whether the patterns of macronutrient changes while maintaining a similar high caloric intake lead to weight cycling and metabolic disturbances. We hypothesized that changes in macronutrients, in addition to the resumption of carbohydrate mixtures with lipids after a low-carbohydrate LCHFD, lead to metabolic changes favoring lipid storage, weight regain and, therefore, weight cycling.

## 2. Materials and Methods

### 2.1. Animals and Experimental Design

All the experiments were performed in accordance with the Guide for the Care and Use of Laboratory Animals published by the US National Institutes of Health (NIH Publication no. 85–23, revised 1996) and were approved by our local ethics committee (CREMEAS, agreement number: 2020111316012887, 13 November 2020).

This study was performed on 30 8-week-old C57BL/6J male mice obtained from ENVIGO (Gannat, France). Mice were housed in groups of two in conventional open-top cages at 22 ± 2 °C on a 12 h day/night cycle and had access to tap water and food ad libitum. For environmental enrichment, cardboard houses, cotton sticks, shredded paper, and wooden chew sticks were placed in the cages to reduce animal stress.

**Figure 1:**
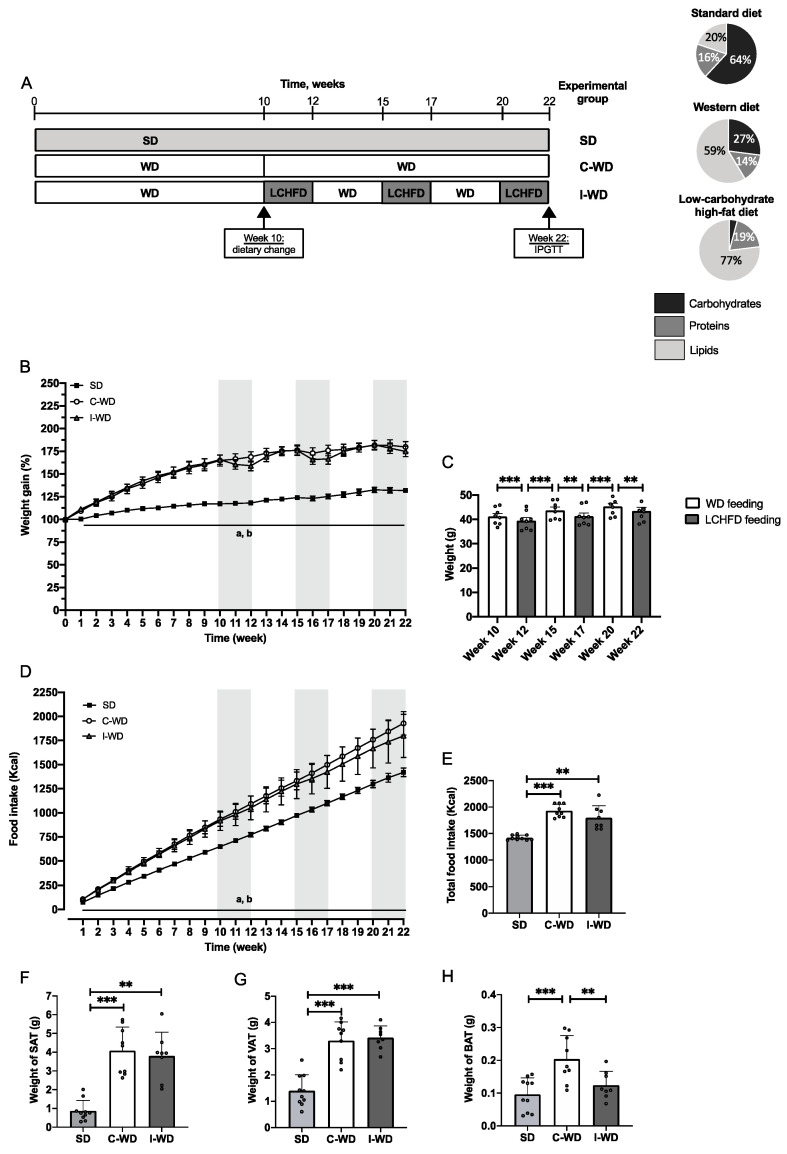
Effect of the C-WD and I-WD on weight gain, food intake and adipose tissue accumulation. (**A**) Experimental design of the procedure, with the SD group (standard diet throughout the study), C-WD group (continuous WD throughout the study), and I-WD group (alternation of 3 periods of 2 weeks with a LCHFD interspersed with 2 periods of 3 weeks with a WD after 10 weeks of a WD) and the macronutrient composition of standard diet (20.5% fat, 15.7% protein and 64% carbohydrate, 3.82 kcal/g), Western diet (58.6% fat, 14.4% protein and 27% carbohydrate, 5.52 kcal/g), and low-carbohydrate, high-fat diet (77% fat, 18.9% protein and 4.2% carbohydrate, 5.55 kcal/g). (**B**). Weight gain throughout the procedure is expressed in grams. For the I-WD group, gray areas represent LCHFD feeding, while white areas represent WD feeding. a = significantly different from the WD group, b = significantly different from the I-WD group. (**C**) I-WD mice were weighed on a gram basis after 3 weeks of being fed the WD (white bars) or after 2 weeks of being fed the LCHFD (gray bars) according to paired *t* tests. (**D**) Food intake in kcal throughout the procedure. For the I-WD group, gray areas represent LCHFD feeding, while white areas represent WD feeding. a = statistically different from the WD group; b = statistically different from the I-WD group. (**E**) Total food intake in kcal. Weight in grams of (**F**) subcutaneous adipose tissue (SAT), (**G**) visceral adipose tissue (VAT), and (**H**) brown adipose tissue (BAT). Mean ± SD (*n* = 8–10). ** *p* < 0.01, and *** *p* < 0.001. a = different from the WD group; b = different from the I-WD group. SD: standard diet, C-WD: continuous Western diet, I-WD: intermittent WD, LCHFD interspersed with WD, LCFHD: low-carbohydrate, high-fat diet.

Obesity was induced by 10 weeks of consumption of a high-fat, high-carbohydrate WD consisting of 58.6% fat, 14.4% protein, 27% carbohydrate, and 5.52 kcal/g (Safe^®^ Diets). Then, the mice were randomly allocated into two groups: (1) the continuous Western diet group (C-WD, *n* = 10), which was fed a WD for 12 weeks and (2) the intermittent western diet group (I-WD, *n* = 10), which was fed 3 periods of a 2-week LCHFD, consisting of 77% fat, 18.9% protein and 4.2% carbohydrate, 5.55 kcal/g (Safe^®^ Diets), followed by 2 periods of 3-week WD. I-WD and C-WD groups were isocaloric, and the mice were compared to the control group (SD, *n* = 10), which was fed a standard chow diet for 22 weeks and consisted of 20.5% fat, 15.7% protein and 64% carbohydrate, 3.82 kcal/g (Safe^®^ Diets). The design of the study is presented in Figure 1A. Body weight and food intake were measured once a week throughout the intervention until the end of the experiment.

### 2.2. Glucose Tolerance Test

Glucose tolerance was assessed after 22 weeks of diet with an intraperitoneal glucose tolerance test (IPGTT) performed after 4 h of fasting. Blood glucose concentrations were measured with a glucose meter (AccuChek Performa, Roche, Basel, Switzerland) on blood samples drawn from the tail vein before injecting 1.5 g/kg glucose and after 0, 15, 30, 45, 60, and 120 min.

### 2.3. Anatomical Measurements and Tissue Samples

Mice were anesthetized in a hermetic cage and ventilated with a mixture of 4% isoflurane (Aerrane, CSP, Cournon, France) and oxygen after 4 h of fasting. Mice were euthanized by cervical dislocation and exsanguinated. The plasma was separated by centrifugation in heparinized tubes and frozen for biochemical analysis. Insulin levels, low-density lipoprotein (LDL), high-density lipoprotein (HDL), total cholesterol, triglycerides (TG), AST, and ALT levels were measured with an AU-480 automated laboratory workstation (Beckman Coulter France SAS, Villepinte, France) by the Institut Clinique de la Souris (Strasbourg, France). The liver, subcutaneous adipose tissue (SAT), visceral adipose tissue (VAT), and brown adipose tissue (BAT) were weighed and snap-frozen in liquid nitrogen for biochemical analysis or fixed in methylbutane and then immersed in liquid nitrogen for subsequent histological analysis.

### 2.4. Liver Histological Analysis

The liver tissue was sectioned at −20 °C on a cryostat microtome after frozen fixation via OCT (10 μm thick, Cryostar NX70, Fisher Scientific, Waltham, MA, USA).

For hematoxylin–eosin (HE) staining, cryosections were fixed in acetone for 3 s and dried at 37 °C. After an hour of drying, the cryosections were stained in Harris’ hematoxylin solution for 2 min, washed in tap water for 3 min, bleached in 1% acid alcohol for 2 s, and then again washed in tap water for 3 min. Then, the cryosections were counterstained in eosin solution for 1 min, washed in tap water for 3 s, in 80% ethanol, in 100% ethanol, and finally mounted with Eukitt medium (Orsatec, Germany).

Cryosections were rehydrated in PBS for 2 min, stained in Oil Red O solution (Sigma-Aldrich, O0625, Burlington, MA, USA) for 3 min, washed in isopropanol (60%) for 30 s, and subsequently washed in deionized water for 1 min. The slides were counterstained by dipping them in Harris’ hematoxylin for 3 min, washed in tap water for 2 min, and mounted in aqueous medium with Aquatex (Sigma-Aldrich, 108635). The stained slides were blinded and captured using a Zeiss Apotome.2 microscope (CTK Instruments, Carlsbad, CA, USA), and lipid accumulation was quantified with Adobe Photoshop (Adobe Systems, San Jose, CA, USA).

### 2.5. RNA Extraction, Reverse Transcription, and Real-Time Quantitative PCR

Gene expression was measured by real-time quantitative PCR. Total liver RNA was isolated using the Magmax mirvana Kit (Applied Biosystems, Waltham, MA, USA) and a KingFisher DuoPrime (Thermo Scientific, Waltham, MA, USA), and quantity and quality were evaluated using a Qubit 4 fluorometer (Thermo Scientific, MA, USA) according to the manufacturer’s instructions. Two micrograms of RNA were reverse-transcribed in a QuantStudio 3 system (Applied Biosystems, MA, USA) to produce cDNA using Maxima H Minus cDNA Synthesis Master Mix (Thermo Scientific, MA, USA). Following the manufacturer’s recommendation, cDNA was amplified in triplicate by real-time PCR in a QuantStudio 3 system in a total reaction volume of 15 μL with PowerTrack™ SYBR Green Master Mix (Applied Biosystems, Foster City, CA, USA). The ΔΔCt method was used to normalize cycle threshold values for each gene of interest, using the hypoxanthine phosphoribosyltransferase (Hprt) gene as a housekeeping gene. Hprt expression was tested and found to be unaffected by the experimental conditions. The primer sets used were designed and obtained from Applied Biosystems (Table 1).

### 2.6. Western Blotting

Liver samples were homogenized in 10 volumes of RIPA buffer (50 mM Tris–HCl (pH 7.5), 150 mM NaCl, 1 mM EGTA, 1 mM EDTA, 100 mM NaF, 5 mM Sodium orthovanadate (Na_3_VO_4_), 1% Triton X-100, 1% sodium dodecyl sulfate (SDS), 40 mM β-glycerophosphate, and protease inhibitor mixture (P8340; Sigma–Aldrich)) using magnetic beads, and centrifuged at 10,000× *g* for 10 min (4 °C). Forty micrograms of protein were loaded into 4–20% SDS–polyacrylamide gels and transferred to nitrocellulose membranes (iBlot 2 Dry Blotting System, Invitrogen, Carlsbad, CA, USA). After 1 h of blocking at room temperature with 50 mM Tris-HCl (pH 7.5), 150 mM NaCl, and 0.1% Tween 20 (TBS-T) containing 5% skim milk, the membranes were incubated with the following primary antibodies directed against fibroblast growth factor 21 (FGF21; Ab Clonal, A3908, 1:1000), total AMPKα (Cell Signaling, Danvers, MA, USA, #2532, 1:1000), phospho AMPKα (Thr172, Cell Signaling, #2535, 1:1000), total ACC1 (Cell Signaling, #3662, 1:1000), phospho-ACC1 (Ser79, Cell Signaling, #3661, 1:1000), PPARγ (Santa Cruz, Sc-150, 1:200), and C/EBPα (Cell Signaling, #8178S, 1:500). After a night of incubation at 4 °C, the membranes were washed three times with TBS-T and incubated for 1 h at room temperature with secondary antibodies directed against rabbit (Cell Signaling, MA, USA; 1:4000 #7074S) or mouse (Cell Signaling, MA, USA; 1:4000 #7076S) antibodies. The proteins were visualized using a Pierce ECL kit (Thermo Fisher Scientific, Waltham, CA, USA) or a SupraSignal Femto kit (Thermo Fisher Scientific, CA, USA) and visualized by enhanced chemiluminescence (iBright 1500 Imaging System, Invitrogen, CA, USA). Ponceau coloration was used as the loading control, and ImageJ software (version 1.8.0) was used for quantification.

### 2.7. Data Analyses

The data are expressed as the mean ± SD, and normally distributed data were checked with the Shapiro–Wilk test. Potential outliers were verified using the ROUT method (1%). Ordinary one-way ANOVA or Kruskal–Wallis tests were used, depending on the normality of the data, and were completed with Holm–Sidak or Dunn’s multiple comparison post hoc tests to explore significant differences between groups. All the statistical analyses were performed using GraphPad Prism 8 (GraphPad Software, Inc., San Diego, CA, USA).

## 3. Results

### 3.1. Changes in Macronutrients during Dieting Induce a Weight-Cycling Pattern and Obesity

Initially, mice in the C-WD and I-WD groups were fed a Western, high-fat, high-sugar diet to induce obesity (Figure 1B). After 10 weeks of WD feeding, a large increase in body weight was induced (Figure 1B), as shown by the large weight gains of 65% and 66% in both C-WD and I-WD mice, respectively, in comparison with those in the SD group (+17%, *p* < 0.001). Between the 10th and 22nd weeks, the C-WD mice continued WD feeding and exhibited a significant increase of +80% in final weight gain in comparison with that of the SD mice (+32%, *p* < 0.001).

After the 10th week, the I-WD mice started the LCHFD interspersed with the WD. At the end of the first LCHFD period (12th week), I-WD mice exhibited weight loss (*p* = 0.0010; Figure 1C), but weight was regained within 3 weeks of resuming the WD (*p* < 0.001; Figure 1C). The same pattern was observed during the second LCHFD/WD cycle, in which I-WD mice experienced weight loss after two weeks of the LCHFD (*p* = 0.0014) but regained their weight within 3 weeks of resuming the WD (*p* = 0.001; Figure 1C). During the last period of the LCHFD, the mice also exhibited significant weight loss (*p* = 0.0042; Figure 1C), but the final weight gain in the I-WD group did not significantly differ from that in the C-WD group (43.4 g versus 45.7 g) and was greater than that in the SD group (*p* < 0.001; Figure 1C). Regarding caloric intake, the C-WD and I-WD mice had similar caloric intakes, which were significantly greater than those of the SD group (*p* < 0.001 and *p* = 0.0067, respectively; Figure 1D,E).

Finally, at the end of the experiment, we measured the weights of the different adipose tissues (AT). Visceral and subcutaneous ATs are involved in fat storage, while brown AT is involved in thermogenesis. The weights of subcutaneous and visceral ATs were greater in the C-WD mice than in the SD mice (*p* < 0.001). There was also a significant increase in both subcutaneous (*p* = 0.0019) and visceral AT (*p* < 0.001) in the I-WD group compared with the SD group (Figure 1F,G). Interestingly, compared with the SD mice, only the C-WD mice exhibited a significant increase in brown adipose tissue (*p* = 0.0010), whereas the I-WD mice maintained a brown adipose tissue weight similar to that of the SD mice (Figure 1H).

### 3.2. Changes in Macronutrients during Dieting Induces Glucose Intolerance and Dyslipidemia

**Figure 2:**
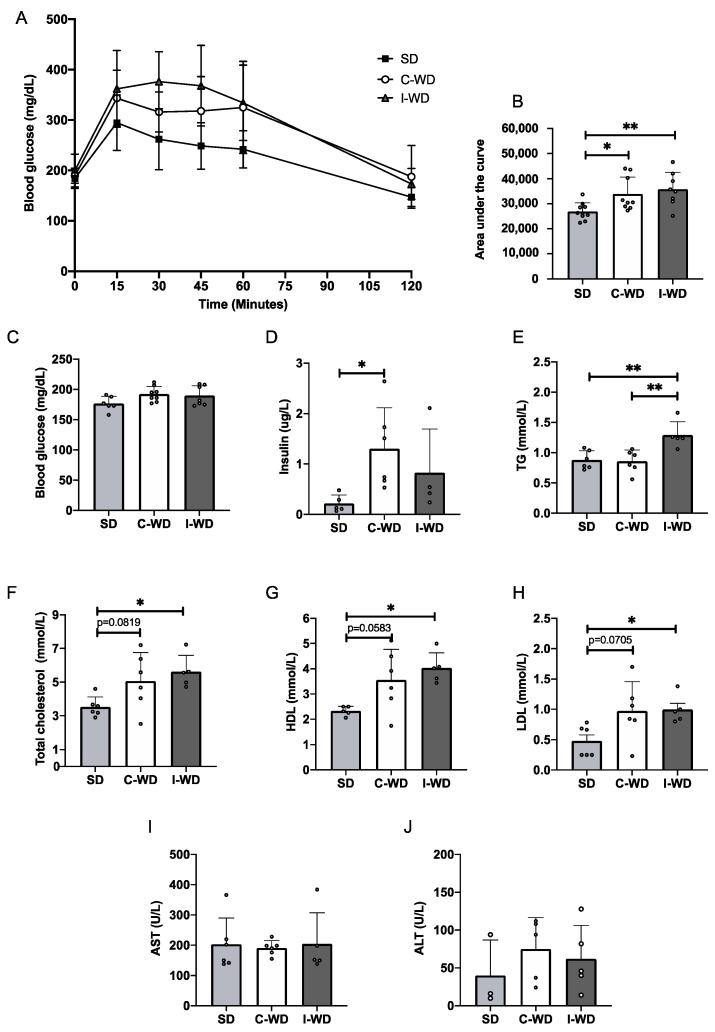
Effects of the C-WD and I-WD on glucose tolerance, insulinemia and lipid profiles. (**A**) IPGTTs were performed at 22 weeks (*n* = 8–10). (**B**) Corresponding area under the curve (*n* = 8–10) for the IPGTT. (**C**) Fasting blood glucose levels measured from the vein tail after 4 h of fasting (*n* = 8–10). (**D**) Fasting insulin levels measured in plasma (*n* = 5–6). Plasma levels of (**E**) total cholesterol (*n* = 5–6), (**F**) high-density lipoprotein (HDL) cholesterol (*n* = 5–6), (**G**) low-density lipoprotein (LDL) cholesterol (*n* = 5–6), (**H**) triglycerides (*n* = 5–6), (**I**) aspartate aminotransferase (AST, *n* = 5–6), and (**J**) alanine aminotransferase (ALT, *n* = 3–5) levels were measured. Mean ± SD. * = *p* < 0.05, and ** = *p* < 0.01. SD: standard diet; C-WD: Continuous western diet; I-WD: intermittent WD; LCHFD interspersed with WD; LCFHD: low-carbohydrate high-fat diet.

After 22 weeks of feeding, neither the C-WD nor the I-WD group exhibited any change in fasting blood glucose levels, but after the administration of glucose, the glycemic response of both the C-WD and I-WD groups was increased in comparison with that of the SD group (+25.7%, *p* = 0.045 and +32.9%, *p* = 0.0081, respectively; Figure 2A–C). Interestingly, only the insulin level of the C-WD mice was significantly greater than that of the SD mice (*p* = 0.0104; Figure 2D).

For the lipid profile (Figure 2E–H), the C-WD mice presented an increasing trend in total cholesterol (*p* = 0.0819), HDL cholesterol (*p* = 0.0582), and LDL cholesterol (*p* = 0.0705) levels in comparison with those of the SD mice, while I-WD mice presented a significant increase in total cholesterol (*p* = 0.0339), HDL cholesterol (*p* = 0.0192), and LDL cholesterol (*p* = 0.0456) levels. Additionally, the triglyceride levels were greater in the I-WD group than in both the SD and C-WD groups (*p* = 0.0052 for both). Finally, neither the C-WD group nor the I-WD group showed any changes in AST or ALT levels compared with the SD group (Figure 2I,J).

### 3.3. Changes in Macronutrients during Dieting Induce Nonalcoholic Steatosis Development

**Figure 3:**
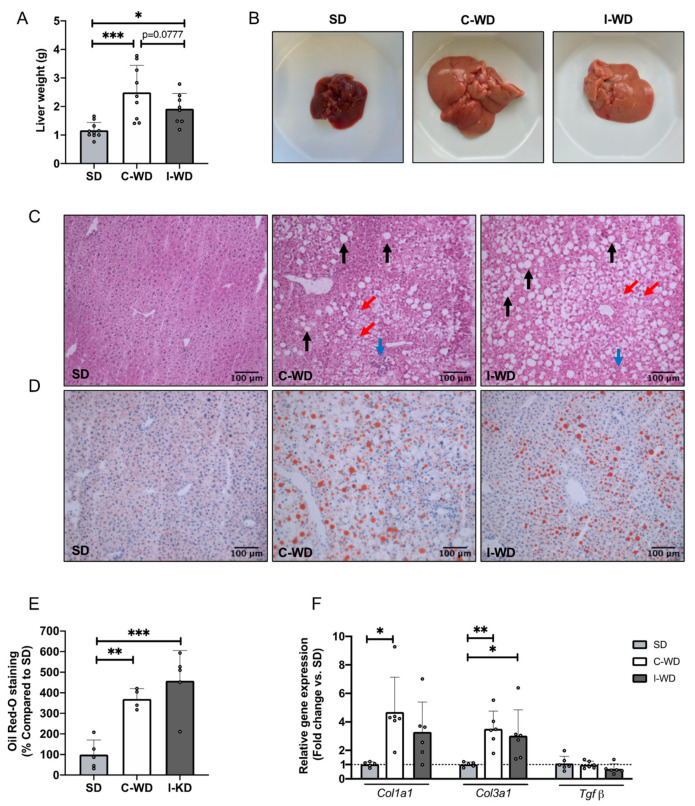
Effects of the C-WD and I-WD on liver histology and relative gene expression of fibrosis markers. (**A**) Liver weight in grams (*n* = 8–10); (**B**) liver gross morphology; (**C**) hematoxylin–eosin staining of liver sections. The black arrows show steatosis, the red arrows show hepatocyte ballooning, and the blue arrows show inflammatory infiltrates. (**D**) Oil Red O staining of liver sections; lipids are stained red. (**E**) Quantification of Oil Red O staining (*n* = 4–5). (**F**) Type I collagen (Col1a1), type III collagen (Col3a1) and transforming growth factor beta (Tgfβ) relative gene expression (*n* = 5–6). Mean ± SD. * = *p* < 0.05, ** *p* < 0.01 and *** *p* < 0.001. SD: standard diet; C-WD: Continuous Western diet; I-WD: Intermittent WD; LCHFD interspersed with WD; LCFHD: low-carbohydrate high-fat diet.

Macroscopic observations revealed a significant increase in liver weight in the C-WD group (+113%, *p* = 0.0004) and in the I-WD group (+64%, *p* = 0.0385) compared with that in the SD group (Figure 3A,B). However, liver weight tended to be lower in the I-WD mice than in the C-WD mice (−29%, *p* = 0.0777). Furthermore, both C-WD and I-WD livers appeared larger and displayed a yellow–brown color, in contrast to the normal dark brown color of SD livers (Figure 3A,B).

At the microscopic level, HE staining revealed widely distributed lipid droplets in the livers of the C-WD and I-WD mice, accompanied by inflammatory infiltration and hepatocyte ballooning (Figure 3C). Oil red O staining revealed a significant increase in hepatic triglyceride levels in C-WD and I-WD livers (*p* = 0.0045 and *p* = 0.0004, respectively) compared with those in SD livers (Figure 3D,E). The presence of significant steatosis, hepatocyte ballooning, and inflammatory infiltrates confirmed the development of NAFLD in the C-WD and I-WD mice [35].

Regarding fibrosis markers, the relative gene expression of type I collagen (Col1a1) and type III collagen (Col3a1) was significantly greater in the C-WD group than in the SD group (+368%, *p* = 0.0063 for type I collagen and +250%, *p* = 0.0219 for type III collagen) (Figure 3E). In addition, the I-WD mice presented a significant increase in collagen gene expression, but only for type III collagen (+202%, *p* = 0.0474). Finally, neither the C-WD nor I-WD affected transforming growth factor β (Tgfβ) gene expression compared to the SD (Figure 3E).

### 3.4. An Intermittent Ketogenic Diet Does Not Activate the FGF21 Signaling Pathway

**Figure 4:**
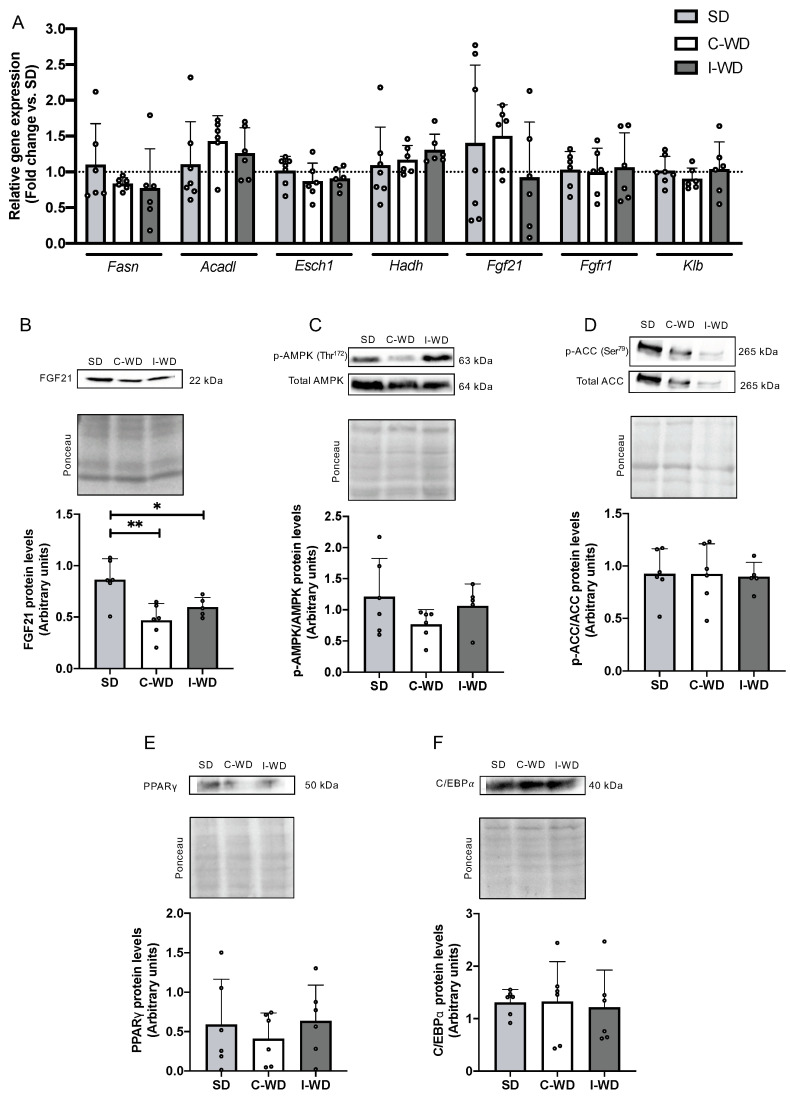
Effect of the C-WD and I-WD on fatty acid metabolism and the FGF21 signaling pathway. Relative gene expression of (**A**) fatty acid synthase (Fasn), acyl-CoA dehydrogenase long chain (Acadl), enoyl-CoA hydratase 1 relative gene expression (Esch1), hydroxyacyl-CoA dehydrogenase (Hadh), fibroblast growth Factor 21 (Fgf21), beta klotho receptor (Klb), and fibroblast growth Factor 21 receptor (Fgfr1). (**B**) FGF21 and fibroblast growth Factor 21 protein levels. (**C**) Ratio of phosphorylated and total AMP-activated protein kinase (AMPK) proteins. (**D**) Ratio of phosphorylated and total acetyl-CoA carboxylase (ACC) levels. (**E**) Peroxisome proliferator-activated receptor gamma protein (PPARγ) levels. (**F**) CCAAT/enhancer-binding protein alpha (C/EBPα) protein levels. Above each panel, Western blots from representative subjects are displayed. Mean ± SD (*n* = 5–6). * *p* < 0.05, ** *p* < 0.01. SD: standard diet; C-WD: continuous Western diet; I-WD: intermittent WD; LCHFD interspersed with WD; LCFHD: low-carbohydrate, high-fat diet.

Finally, we explored the effect of changes in macronutrients on liver metabolism, particularly by examining the metabolic pathways usually regulated by an LCHFD, including the fatty acid oxidation and fibroblast growth Factor 21 (FGF21) pathways. C-WD and I-WD mice did not differ from SD mice in terms of the expression of fatty acid synthase (Fasn), the beta-oxidation genes acyl-CoA dehydrogenase long-chain (Acadl), enoyl-CoA hydratase 1 (Esch1), and hydroxyacyl-CoA dehydrogenase (Hadh), or in the expression of Fgf21, fibroblast growth factor receptor 1 (Fgfr1), and its co-receptor β-klotho (Klb) (Figure 4A).

Interestingly, at the protein level, the C-WD and I-WD groups exhibited significant decreases in FGF21 (−46%, *p* = 0.0028 and −31%, *p* = 0.034, respectively) compared with the SD group (Figure 4B). Moreover, neither C-WD nor I-WD mice exhibited any changes in AMPK or ACC phosphorylation or in PPARγ or C/EBPα levels (Figure 4C–F).

## 4. Discussion

Our study explored the consequences of repeated dietary shifts without caloric restriction, alternating between an LCHFD and a WD (I-WD). We demonstrated that these dietary changes resulted in weight loss during each LCHFD cycle, while reverting to the WD led to significant weight regain, ultimately contributing to the development of severe obesity and adipose tissue accumulation. The I-WD mice exhibited an increase in the glucose tolerance test curve, highlighting the development of glucose intolerance, and an increase in total cholesterol, LDL, HDL, and TG levels, indicating dyslipidemia [36]. In the liver, the I-WD mice exhibited hepatomegaly development, significant steatosis associated with hepatocyte ballooning, and type 3 collagen gene expression, which are distinctive of NAFLD development [35,37]. This pattern of repetitive weight loss and regain associated with the development of metabolic complications is characteristic of weight cycling [5]. In the I-WD mice, the consumption of a high-fat but low-carbohydrate hypercaloric diet led to weight loss but the transition to a high-fat, high-sugar WD was enough to induce complete weight regain and hepatic lipid accumulation, even though the WD and LCHFD were isocaloric. These results were quite surprising, challenging the main hypothesis that explained the development of weight cycling during a diet, which focused on metabolic adaptations in response to fluctuations in caloric intake [25,26].

Our findings shed light on weight-cycling establishment, indicating that caloric intake alone may not be the sole determinant. Instead, the macronutrient composition of the diet, particularly the reintroduction of carbohydrates, appears to play a significant role in weight regain. Notably, numerous long-term studies on low-carbohydrate, high-fat diets (LCHFD) have demonstrated weight loss despite the high caloric consumption [27,28]. However, our study reveals that transitioning from an LCHFD to a Western diet (WD) under isocaloric conditions also results in considerable weight regain, suggesting that the trigger for weight cycling lies in the return to a WD [29]. The impact of carbohydrates on weight gain has previously been suggested to be a central element in the development of obesity. The carbohydrate–insulin model of obesity theorizes that the accumulation of adipose tissue is due not only to a high caloric intake but also to the propensity of carbohydrates to increase insulin secretion and thus stimulate de novo lipogenesis [38,39]. Our results demonstrated increased adiposity in both subcutaneous and visceral tissue as well as in the liver, suggesting the activation of de novo lipogenesis in the I-WD mice. We hypothesize that fluctuations in insulin levels in response to shifts in macronutrient intake may contribute to the development of weight cycling, thus explaining the weight regain phenomenon. Several studies have shown that LCHFD consumption significantly decreases insulin levels while WD consumption is associated with obesity development and high insulin levels [27,29,40]. Therefore, the return of a high-fat, high-sugar WD after LCHFD-induced weight loss could lead to hyperinsulinemia and promote weight regain. However, our results do not allow us to come to conclusions about insulin secretion variations during diet changes because insulin was measured only at the end of the procedure. Further studies should assess the kinetics of insulin fluctuations during I-WD feeding to evaluate the role of insulin in weight regain and its impact on de novo lipogenesis in the liver.

Indeed, in the liver, for I-WD mice, an increase in liver weight, a hepatomegaly, and a significant steatosis were found, showing the development of NAFLD. At the molecular level, the I-WD mice showed an increase in Col3a1 gene expression, suggesting an activation of the fibrotic process in the liver [41]. If the increase in steatosis in the I-WD group might be a consequence of the lipid intake increase during the periods of an LCHFD, several pre-clinical and clinical studies showed that an LCHFD did not induce hepatic fat accumulation, and had beneficial effects on NAFLD [28,33,42,43]. Moreover, the development of NAFLD induced by weight cycling has already been observed in other studies, which suggest that the increase in the risk of hepatic complications is due to the weight fluctuations [44,45]. The mechanisms behind the link between weight cycling and NAFLD remain unclear but could involve a redistribution of body fat towards visceral adipose tissue, strengthening the risk of metabolic complications, including NAFLD [10]. Moreover, an increase in blood sugar and, therefore, insulin levels after resuming a high-carbohydrate diet could activate hepatic fatty acid storage by promoting de novo lipogenesis process [46]. However, I-WD mice did not exhibit any variation in Fasn gene expression or phosphorylated ACC, PPARγ, or C/EBPα protein levels, though these targets are involved in the fatty acid storage pathway [47]. In the WD diet, both macronutrients contribute to the synthesis and storage of triglycerides in the liver. The high carbohydrate content leads to elevated insulin levels, which promote de novo lipogenesis, while the high-fat content provides an additional source of fatty acids, which can be esterified with glycerol to form triglycerides [48]. This dual impact of both carbohydrates and fats on hepatic triglyceride synthesis can contribute to the development of fatty liver disease in individuals consuming this type of diet. Therefore, the large accumulation of triglycerides in adipose and liver tissues strongly suggested that de novo lipogenesis and TG synthesis activation occurred throughout the procedure, probably in earlier stages, and reinforced the potential role of insulin rising after the resumption of a WD and weight regain in NAFLD development.

Interestingly, weight cycling not only seems to affect visceral and subcutaneous white adipose tissues by increasing their accumulation but also seems to impact brown adipose tissue. Indeed, while the C-WD mice presented an increase in BAT, the BAT levels found in I-WD mice were similar to those in SD mice. BAT hyperplasia is commonly reported in obese rodents overfed with a WD and contribute to energy expenditure in response to chronic hypercaloric intake by the dissipation of chemical energy as heat [49,50]. Therefore, the decrease in BAT in the I-WD mice compared to the C-WD mice could be a consequence of weight cycling and could act as a mechanism to resist weight loss by decreasing heat-loss-related energy expenditure [22,51]. Indeed, a decrease in BAT reduces the resting metabolic rate to decrease energy expenditure through heat loss in brown adipose tissue [52,53]. A study conducted on BAT-deprived mice fed a WD demonstrated that the absence of BAT enhances weight gain and metabolic complications, suggesting that BAT plays a role not only in energy expenditure but also in energy savings [54]. Therefore, we hypothesize that when caloric intake is high, the weight loss phases induced by an LCHFD lead to metabolic adaptations aiming to minimize energy expenditure, ultimately facilitating weight regain upon returning to a WD and, thus, weight cycling. Complementary studies exploring BAT activity should be performed to determine whether a decrease in BAT is a cause or a consequence of the development of weight cycling. Finally, we also found a decrease in the protein level of FGF21, which is a hormone known to be a major regulator of energy homeostasis by increasing lipid catabolism and reducing lipogenesis [55] in both the I-WD and C-WD mice. The phenotypes observed in both the C-WD and I-WD mice, characterized by obesity development, steatosis, and liver alterations, were also found in FGF21 knockout mice [56,57]. These results may suggest that a reduction in FGF21 can participate in the progression of obesity in both WD and I-WD mice. The decrease in FGF21 was unexpected in the I-WD group, as an LCHFD usually strongly increases the FGF21 gene and protein expression [28,29]. These findings imply that weight cycling could lead to FGF21 disturbance and promote the development of metabolic complications. However, the regulatory effect of FGF21 is complex, influenced by many factors, and not fully understood. Additional work could be carried out to clarify its role in weight gain and obesity development and the impact of weight cycling on FGF21 kinetics.

Contrary to previous assumptions suggesting that weight cycling is primarily driven by fluctuations in caloric intake during dieting, our study reveals a different scenario. We found that alterations in macronutrient consumption, rather than a reduction in overall caloric intake, were associated with weight cycling. This sheds new light on our understanding of the phenomenon, proposing that weight loss maintenance should not solely be viewed through the lens of calorie management but also through macronutrient composition. Our results also emphasized the harmful impact of carbohydrate reintroduction, which leads to significant weight regain and is potentially linked to increased insulin levels. Interestingly, despite evolving nutritional perspectives, numerous government guidelines continue to endorse a daily carbohydrate intake ranging from 40% to a maximum of 75% to achieve a healthy eating pattern [58]. Our findings are particularly important for the management of patients with obesity because they demonstrate that the macronutrient composition of diets should also be strictly controlled during weight loss management to prevent the establishment of weight cycling and to avoid the risk of aggravating metabolic complications. Considering our findings, numerous studies should be conducted regarding the significant consumption of carbohydrates to better understand the acceptable sugar quality in the diet to avoid the risk of weight cycling and the potential development of symptoms indicating progression toward steatosis or even type 2 diabetes.

## 5. Conclusions

Our study revealed that caloric intake is not the only trigger for weight cycling. We demonstrated that the return to a high-fat, high-sugar WD after LCHFD-induced weight loss leads to weight cycling and metabolic complications, similar to what was observed in the obese WD group, showing that dietary changes from an LCHFD to a WD are at least as deleterious as a long-term WD. Our results suggest that the resumption of carbohydrates and sugar is responsible for weight cycling in I-WD mice. On the other hand, the molecular pathways involved in the establishment of weight cycling patterns have not been identified but could include variations in insulin activation pathways. Further studies will be necessary to understand the relationships between dietary changes, insulin levels, and metabolic adaptations that lead to weight cycling and the development of complications. Despite the positive effects of the LCHFD, returning to a Western diet poses the risk of losing these benefits and potentially triggering weight cycling. Therefore, there is a need to reduce sugar intake and establish sustainable long-term dietary habits.

## Figures and Tables

**Table 1:** Primers used for liver qPCR.

*Gene*	*Forward Primer*	*Reverse Primer*
*Hprt*	GTTGGATACAGGCCAGACTTTGTTG	GATTCAACTTGCGCTCATCTTAGGC
*Acadl*	GAAGATGTCCGATTGCCAGC	AGTTTATGCTGCACCGTCTGT
*Col1a1*	GACCGTTGAGTCCGTCTTTG	TCATCGTGGCTTCTCTGGCT
*Col3a1*	CTGGCCCTCCTGGTGCTTCT	CCTTGGCCCATCCTTTCCTG
*Esch1*	GCAAAGCAGGCAGGTCTTGT	TAGCTGCCAGTTCTCAGTGG
*Hadh*	TCGTGAACCGACTCTTGGTG	ATTTCATGCCACCCGTCCAA
*Fasn*	TGCACCTCACAGGCATCAAT	GTCCCACTTGATGTGAGGGG
*Fgf21*	GTGTCAAAGCCTCTAGGTTTCTT	GGTACACATTGTAACCGTCCTC
*Fgfr1*	GCCAGACAACTTGCCGTATG	ATTTCCTTGTCGGTGGTATTAACT
*Klb*	GGACACAACCTGATCAAGGCAC	GAGAACTCGGGGATCATGGC
*Tgf β*	ACGTGGAAATCAACGGGATCA	GTTGGTATCCAGGGCTCTCC

Hprt, Hypoxanthine phosphoribosyltransferase. Acadl, acyl-CoA dehydrogenase long chain. Col1a1, collagen type I alpha 1 chain. Col3a1, collagen type III alpha 1 chain. Esch1, enoyl-CoA hydratase 1. Hadh, hydroxyacyl-CoA dehydrogenase. Fasn, Fatty acid synthase. Fgf21, fibroblast growth Factor 21. Fgfr1, fibroblast growth factor receptor 1. Klb, β-Klotho receptor. Tgfβ, transforming growth factor beta.

## Data Availability

The original contributions presented in the study are included in the article. Further inquiries can be directed to the corresponding author.
